# Spinal Cord Stimulation Exerts Neuroprotective Effects against Experimental Parkinson’s Disease

**DOI:** 10.1371/journal.pone.0101468

**Published:** 2014-07-10

**Authors:** Aiko Shinko, Takashi Agari, Masahiro Kameda, Takao Yasuhara, Akihiko Kondo, Judith Thomas Tayra, Kenichiro Sato, Tatsuya Sasaki, Susumu Sasada, Hayato Takeuchi, Takaaki Wakamori, Cesario V. Borlongan, Isao Date

**Affiliations:** 1 Department of Neurological Surgery, Okayama University Graduate School of Medicine, Okayama, Japan; 2 Department of Neurosurgery, University of South Florida College of Medicine, Tampa, Florida, United States of America; Hospital General Dr. Manuel Gea González, Mexico

## Abstract

In clinical practice, deep brain stimulation (DBS) is effective for treatment of motor symptoms in Parkinson’s disease (PD). However, the mechanisms have not been understood completely. There are some reports that electrical stimulation exerts neuroprotective effects on the central nervous system diseases including cerebral ischemia, head trauma, epilepsy and PD, although there are a few reports on neuroprotective effects of spinal cord stimulation (SCS). We investigated the neuroprotective effects of high cervical SCS on PD model of rats. Adult female Sprague-Dawley rats received hour-long SCS (2, 50 or 200 Hz) with an epidural electrode at C1–2 level for 16 consecutive days. At 2 days after initial SCS, 6-hydroxydopamine (6-OHDA) was injected into the right striatum of rats. Behavioral evaluations of PD symptoms were employed, including cylinder test and amphetamine-induced rotation test performed at 1 and 2 weeks after 6-OHDA injection. Animals were subsequently euthanized for immunohistochemical investigations. In order to explore neurotrophic and growth factor upregulation induced by SCS, another cohort of rats that received 50 Hz SCS was euthanized at 1 and 2 weeks after lesion for protein assays. Behavioral tests revealed that the number of amphetamine-induced rotations decreased in SCS groups. Immunohistochemically, tyrosine hydroxylase (TH)-positive fibers in the striatum were significantly preserved in SCS groups. TH-positive neurons in the substantia nigra pars compacta were significantly preserved in 50 Hz SCS group. The level of vascular endothelial growth factor (VEGF) was upregulated by SCS at 1 week after the lesion. These results suggest that high cervical SCS exerts neuroprotection in PD model of rats, at least partially by upregulation of VEGF. SCS is supposed to suppress or delay PD progression and might become a less invasive option for PD patients, although further preclinical and clinical investigations are needed to confirm the effectiveness and safety.

## Introduction

Parkinson’s disease (PD) is a progressive neurodegenerative disease caused by the loss of dopaminergic neurons in the nigrostriatal system. Levodopa treatment remains the gold standard of treatment for PD, but only supportive effects are achieved with adverse effects (e.g., dyskinesias) over time [1] necessitating the need for innovative therapies.

In surgical treatment, deep brain stimulation is effective for motor symptoms of PD, but the inclusion criteria have been limited. In 2009, Fuentes and colleagues reported that spinal cord stimulation (SCS) restores locomotion in animal models of PD [2]. In the clinic, SCS is an alternative approach for treatment of neuropathic pain after medication has failed. Previously, laboratory studies have shown that SCS increases cerebral blood flow in animal models of cerebral ischemia or spasms after subarachnoid hemorrhage through vasodilation effects with subsequent behavioral amelioration [3]–[10], and recently, a study demonstrated that SCS at the high thoracic level for PD model of rats has neuroprotective effects [11].

Neuroprotective effects of electrical stimulation have been demonstrated in our preclinical reports [12], [13], but the mechanisms of action remain incompletely understood. In ischemic stroke rats, neurotrophic factors (glial cell line-derived neurotrophic factor: GDNF; brain-derived neurotrophic factor: BDNF), and vascular endothelial growth factor (VEGF) were upregulated by cortical stimulation [12]. Moreover, in PD rats, stimulation of the subthalamic nucleus generated neuroprotective effects [14]–[17], with BDNF implicated as a therapeutic target for the observed neuroprotection [16]. In the present study, we characterized behaviorally and immunohistochemically neuroprotective effects of high cervical SCS on PD rats, with emphasis on the likely involvement of secretion of neurotrophic and growth factors as a key mechanism of action.

## Materials and Methods

### Ethics statement

All animal procedures in this study were specifically approved by the Institutional Animal Care and Use Committee of Okayama University Graduate School of Medicine (protocol #OKU-2012311).

### Animals

Adult female Sprague-Dawley rats (Charles River, Japan; n = 80) weighing 200–250 g at the beginning of the experiment were used. They were singly housed per cage in a temperature and humidity-controlled room, maintained on a 12-hour light/dark cycle, with free access to food and water.

### Experimental design

In order to evaluate the neuroprotective effects of high cervical SCS, rats were classified into 4 groups, namely, control group and 2, 50 and 200 Hz SCS groups respectively (total 40 rats, each group n = 10). All rats received the lower half of C1 and C2 laminectomy under general anesthesia with implantation of a monopolar electrode. We selected the high cervical cord as a target, because we expected that the high cervical SCS might activate upper level cord and brain stem. Subsequently rats in SCS groups received hour-long electrical stimulations daily for 16 days, but rats in control group received no electrical stimulation. At 2 days after initial SCS, 6-hydroxydopamine (6-OHDA) was injected into the right striatum of all rats as described previously (see below). We started SCS at 2 days before 6-OHDA administration, because we expected the pre-conditioning effect of SCS, and stimulation continued for 2 weeks (1 hour/day) after 6-OHDA administration in which degeneration of dopaminergic neurons is going on. For behavioral evaluations of PD symptoms, cylinder test was performed at 1 and 2 weeks after 6-OHDA injection and amphetamine-induced rotation test was performed at 2 weeks after the lesion. In detail, we conducted cylinder test just before stimulation at 1 week after 6-OHDA administration. At 2 weeks after 6-OHDA administration, cylinder test and amphetamine-induced rotation test in twelve hours after the last SCS to minimize the effects of anesthetic.

**Figure 1:**
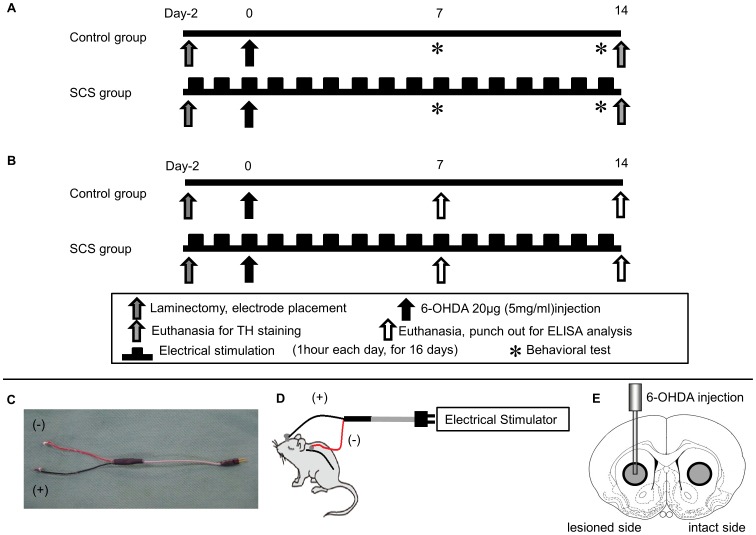
Time course and SCS electrode, and the brain region punched out for protein assay. (A) Scheme showing overall experimental design. (B) Scheme showing experimental design for protein assay. (C) Photograph showing SCS electrode used in this study (diameter: 2 mm; wire length: 60 mm). (D) Scheme showing a rat during stimulation. (E) Brain tissue (diameter: 3 mm showing gray circle), corresponding to the striatum, was punched out from both the lesioned and the intact side.

Following behavioral tests, animals were euthanized for immunohistochemical investigations. In order to evaluate the relationship between SCS and secretion of neurotrophic and growth factors, another cohort of rats was randomly assigned to one of two groups (total 40 rats: control group and 50 Hz SCS group, n = 20, respectively) using the corresponding procedures as described above. These experimental designs are shown in Fig. 1A and B.

### Implantation of an electrode

All rats were deeply anesthetized with pentobarbital (35 mg/kg, i.p.) and placed in a stereotaxic instrument (Narishige, Japan). Laminectomy of the lower half of C1 and C2 was performed. A silver ball electrode with diameter of 2 mm was implanted in the epidural space over the dorsal column. A ground electrode was placed in the skull of rats with the connection side of the electrode outside the body through the subcutaneous lead. The systems used in this study are shown in Fig. 1C and D.

### Electrical stimulation

Rats in both control group and SCS groups were anesthetized with pentobarbital (10 mg/kg, i.p.) with subsequent connection of the electrode to the stimulation device (SEN-7203, NIHON KOHDE, Japan). Thereafter, rats in SCS groups received hour-long stimulations daily for 16 days. To minimize the effects of anesthesia, we used a very small amount of anesthetic, so rats were immobilized only during stimulation. We also used the 26 G thin needle for injection.

The parameter of stimulating pulses was adjusted to a variety of frequency (2, 50, or 200 Hz) based on the results of our previous studies and another basic reports demonstrating therapeutic efficacy [2], [4], [7], [12], and intensities were individually adjusted according to the 80% motor threshold intensity. After the stimulation, the electrode connection was removed, and rats were allowed to move freely.

### 6-OHDA lesion

All rats were anesthetized with sodium pentobarbital (35 mg/kg, i.p.) and placed in a stereotaxic instrument (Narishige, Japan). Twenty µg of 6-OHDA (4 µl of 5 mg/ml dissolved in saline containing 0.2 mg/ml ascorbic acid; Sigma, USA) was injected into the right striatum with a 28 G Hamilton syringe. The lesion coordinates were as follows: 1 mm anterior to the bregma, 3 mm lateral to the sagittal suture, and 5 mm ventral to the surface of the brain with the tooth-bar set at −3.0 mm [18]. The injection rate was 1 µl/min. After the injection, the syringe was left in place for additional 5 minutes before being it was retracted slowly (1 mm/min).

### Behavioral tests

#### Cylinder test

We performed the cylinder test, which assessed the degree of forepaw asymmetry, at 1 and 2 weeks after 6-OHDA injection. Rats were placed in a transparent cylinder (diameter: 20 cm, height: 30 cm) for 3 minutes and the number of forepaw contacts to the cylinder wall was counted [19]. The score of cylinder test in this study was calculated as a contralateral bias, that is, [(the number of contacts with the contralateral limb)-(the number of contacts with the ipsilateral limb)/(the number of total contacts) ×100] [20].

#### Amphetamine-induced rotation test

All rats were tested with amphetamine (3.0 mg/kg, Dainippon Sumitomo Pharma, Japan) at 2 weeks after 6-OHDA injection. The rotational behaviors were assessed for 90 minutes with a video camera. Full 360° turns ipsilateral to the lesion were counted.

### Immunohistochemical investigations

All rats were euthanized with an overdose of pentobarbital (100 mg/kg) at 2 weeks after 6-OHDA injection, and perfused transcardially with 200 ml of cold PBS and 200 ml of 4% paraformaldehyde (PFA) in PBS. Brains were removed and post-fixed in the same fixative overnight at 4 degrees C, and subsequently stored in 30% sucrose in PBS until completely submerged. The brains were coronally sectioned at the thickness of 40 µm. Free-floating sections for TH staining were blocked by 3% hydrogen peroxide in 70% methanol for 7 minutes. Sections were washed 3 times for 5 minutes in PBS. Sections were then incubated overnight at 4 degrees C with rabbit anti-TH antibody (1∶500; Chemicon, Temecula, CA, USA) with 10% normal horse serum. After several rinses in PBS, sections were incubated for 1 hour in biotinylated donkey anti-rabbit IgG (1∶500; Jackson Immuno-Research Lab, West Grove, PA, USA), then for 30 minutes in avidin-biotin-peroxidase complex (Vector Laboratories, Burlingame, CA, USA). Subsequently, the sections were treated with 3, 4-diaminobenzidine (DAB; Vector) and hydrogen peroxide, mounted on albumin-coated slides and embedded with cover glass.

### Morphological analyses

The density of TH-positive fibers in the striatum of rats was determined and analyzed with a computerized analysis system as described previously [21]. Five sections at 0.5±1.0 mm anterior to the bregma were randomly selected for quantitative analyses [18]. The two areas adjacent to the needle tract of lesioned side and the symmetrical areas in the contralateral side were analyzed, respectively. The percentages of lesion to the intact side were evaluated in each section and the averages were used for statistical analyses. The images were computer-processed into binary images using an appropriate threshold (Scion Image, Scion Corp., Frederick, MD, USA). The areas were then calculated and used for statistical analyses. According to our previous publication for counting the number of TH-positive neurons [22], every fifth 40 µm-thick coronal section through the substantia nigra pars compacta (SNc) was explored using 3 coronal sections, respectively, at 4.8, 5.3, and 5.8 mm posterior to the bregma. The number of cells was summed up in each group. The percentage to the intact side was analyzed and the average was used for the statistical analyses.

### ELISA Analyses

For protein assay, fresh brains from rats of control and 50 Hz SCS groups were quickly harvested after decapitation of animals anesthetized with an overdose of pentobarbital (100 mg/kg, i.p.) at 1 and 2 weeks after 6-OHDA lesion. Brains were sliced at the thickness of 2 mm. The brain tissue of the striatum was punched out using a biopsy punch (3 mm-hole, Kai corporation and Kai industries co., ltd, Japan) as shown in Fig. 1E. Brain tissues were then homogenized in T-PER (Pierce, Rockfold, IL) and centrifuged at 10,000 G for 10 minutes at 4 degrees C, and the supernatant was obtained. Brain VEGF and GDNF levels were measured by the usage of rat VEGF ELISA assay kit (IBL, Japan) and rat GDNF ELISA assay kit (Abnova, Taiwan).

### Statistical Analyses

Cylinder test data were evaluated statistically using repeated measures of ANOVA (analysis of variance), while the data from amphetamine-induced rotation test, immunohistochemistry, and ELISA were evaluated statistically using single ANOVA, with subsequent post hoc Scheffe’s test. Statistical significance was preset at p<0.05. Mean values are presented with standard deviation (SD).

## Results

### Behavioral tests

#### Cylinder test

**Figure 2:**
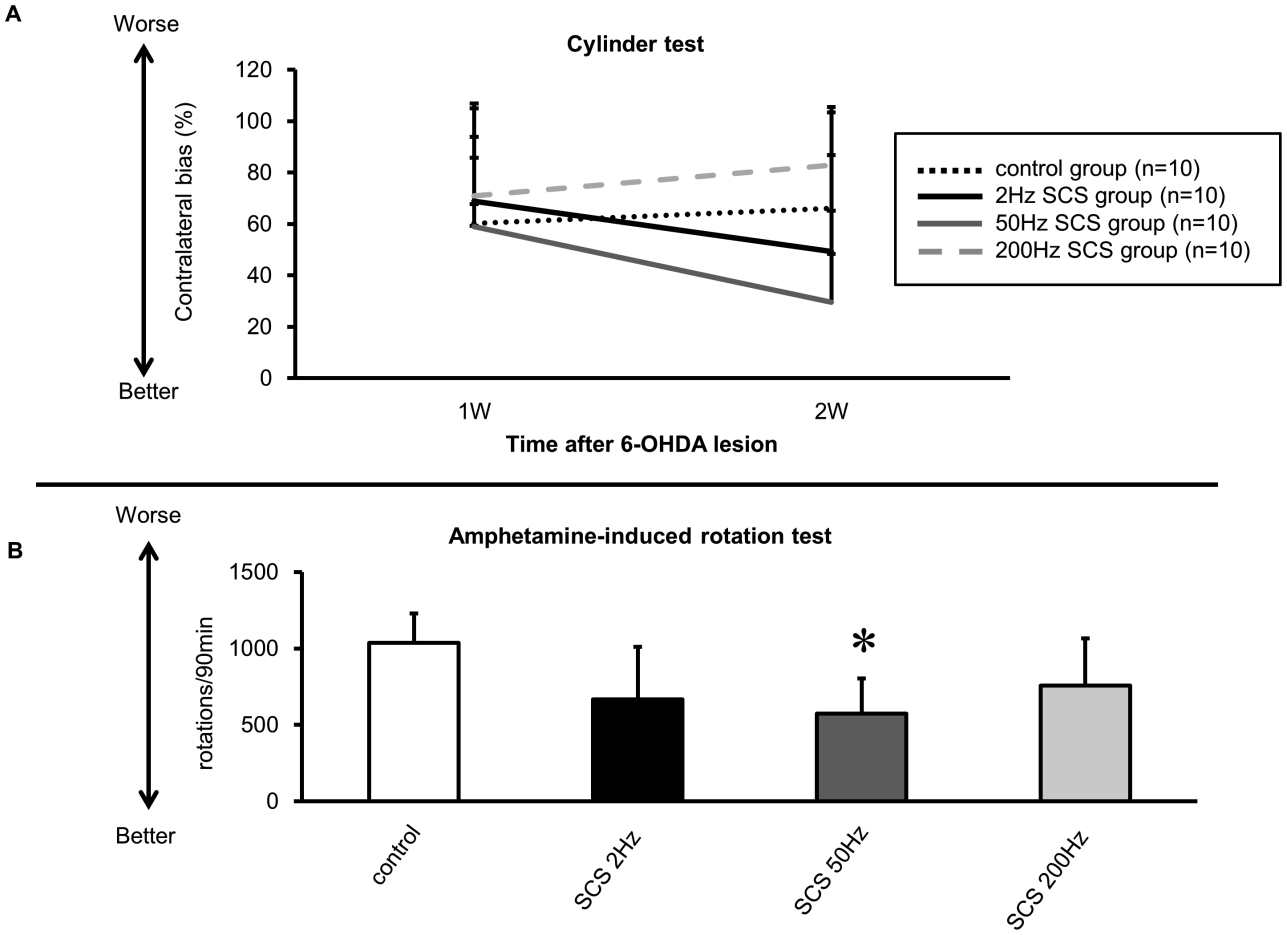
The results of cylinder test and amphetamine-induced rotation test. (A) Rats receiving 2 Hz and 50 Hz SCS showed reduction of the contralateral bias at 2 weeks after 6-OHDA lesion, compared to that of rats in control group. (B) The number of amphetamine-induced rotations in all SCS groups decreased, compared to that of control group. There was a significant amelioration in 50 Hz SCS group, compared to control group (*p<0.05, n = 10, respectively).

In 2 Hz and 50 Hz SCS groups, the treated animals appeared to perform better in the cylinder test than those in 200 Hz SCS and control groups, but did not reach statistical significance at 1 and 2 weeks after 6-OHDA injection (Contralateral bias: 2 Hz: 69±38.0 and 49±54.1%; 50 Hz: 59±46.0 and 29±35.6%; 200 Hz: 71±23.1 and 83±22.5%; control group: 60±25.4 and 66±20.7%, at 1 and 2 weeks respectively; repeated-measures ANOVA; F_(3, 29)_ = 1.871, p = 0.1566) (Fig. 2A).

#### Amphetamine-induced rotation test

The number of amphetamine-induced rotations at 2 weeks after 6-OHDA injection in animals exposed to 2 Hz, 50 Hz, and 200 Hz SCS (2 Hz: 667±344 turns/90 min; 50 Hz: 575±230 turns/90 min; 200 Hz: 759±307 turns/90 min respectively, Fig. 2B) decreased compared to control group (1037±192 turns/90 min, Fig. 2B). In addition, the number of amphetamine-induced rotations significantly decreased in the other cohort of animals exposed to 50 Hz SCS compared to that in control group (ANOVA; F _(3, 32)_ = 5.212; p = 0.0048; p value<0.05).

### Immunohistochemical investigations

**Figure 3:**
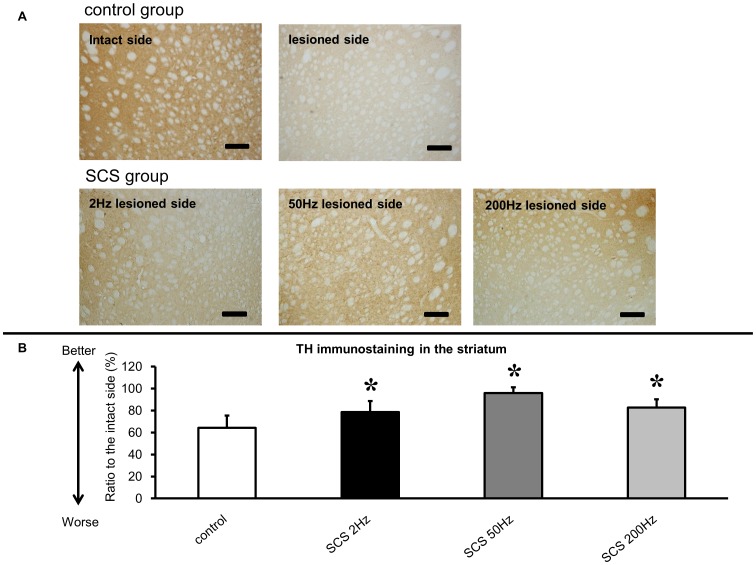
Tyrosine hydroxylase (TH) immunostaining in the striatum and the ratio to the intact side. (A) TH immunostaining in the striatum. Severe loss of TH-positive fibers was seen in the lesioned striatum of control group. Preservation of TH-positive fibers was seen in the lesioned striatum of all SCS groups. Scale bar: 200 µm. (B) The all SCS groups showed significant preservation of TH-positive fibers in the lesioned striatum, compared to those in control group (*p<0.05, n = 10, respectively).

Rats in all SCS groups showed significant preservation of TH-positive fibers in the striatum (2 Hz: 78±9.9%; 50 Hz: 96±5.2%; 200 Hz: 83±7.5% relative to the intact side, respectively, Fig. 3), compared to those in control group (64±11.1%, ANOVA; F_(3, 33)_ = 20.731; p<0.0001; p value<0.05, Fig. 3).

**Figure 4:**
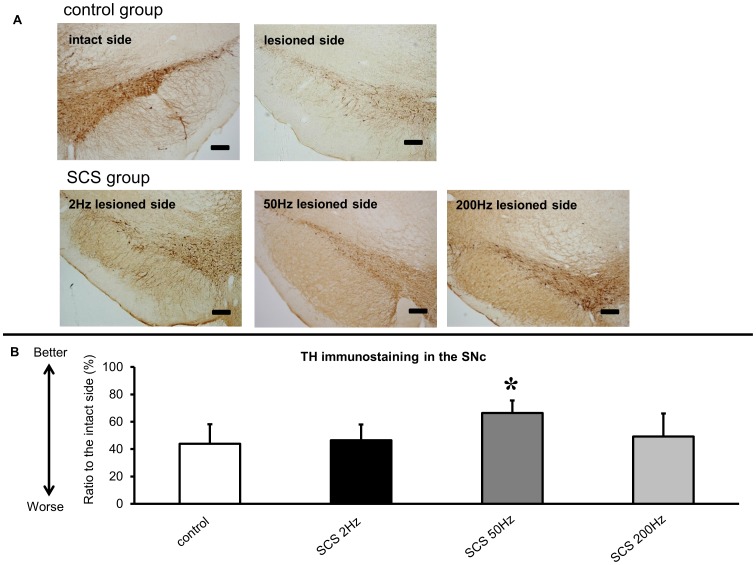
TH immunostaining in the substantia nigra pars compacta (SNc), and the ratio to the intact side. (A) TH immunostaining in the intact SNc. Severe loss of TH-positive neurons was seen in the lesioned side SNc of control group. Preservation of TH-positive neurons was seen at the lesioned side SNc of 50 Hz SCS group. Scale bar: 200 µm. (B) Significant preservation of TH-positive neurons in the lesioned-side SNc of 50 Hz SCS group, compared to those of control group (*p<0.05, n = 10, respectively).

The rats that received 50 Hz SCS also displayed significant preservation of TH-positive neurons in the SNc (66±9.2% for 50 Hz SCS group relative to the intact side, ANOVA; F_(3, 31)_ = 5.155; p = 0.0052; p value<0.05, Fig. 4), compared to those in control group (44±14.4% relative to the intact side, Fig. 4).

### Protein assay for neurotrophic and growth factors

**Figure 5:**
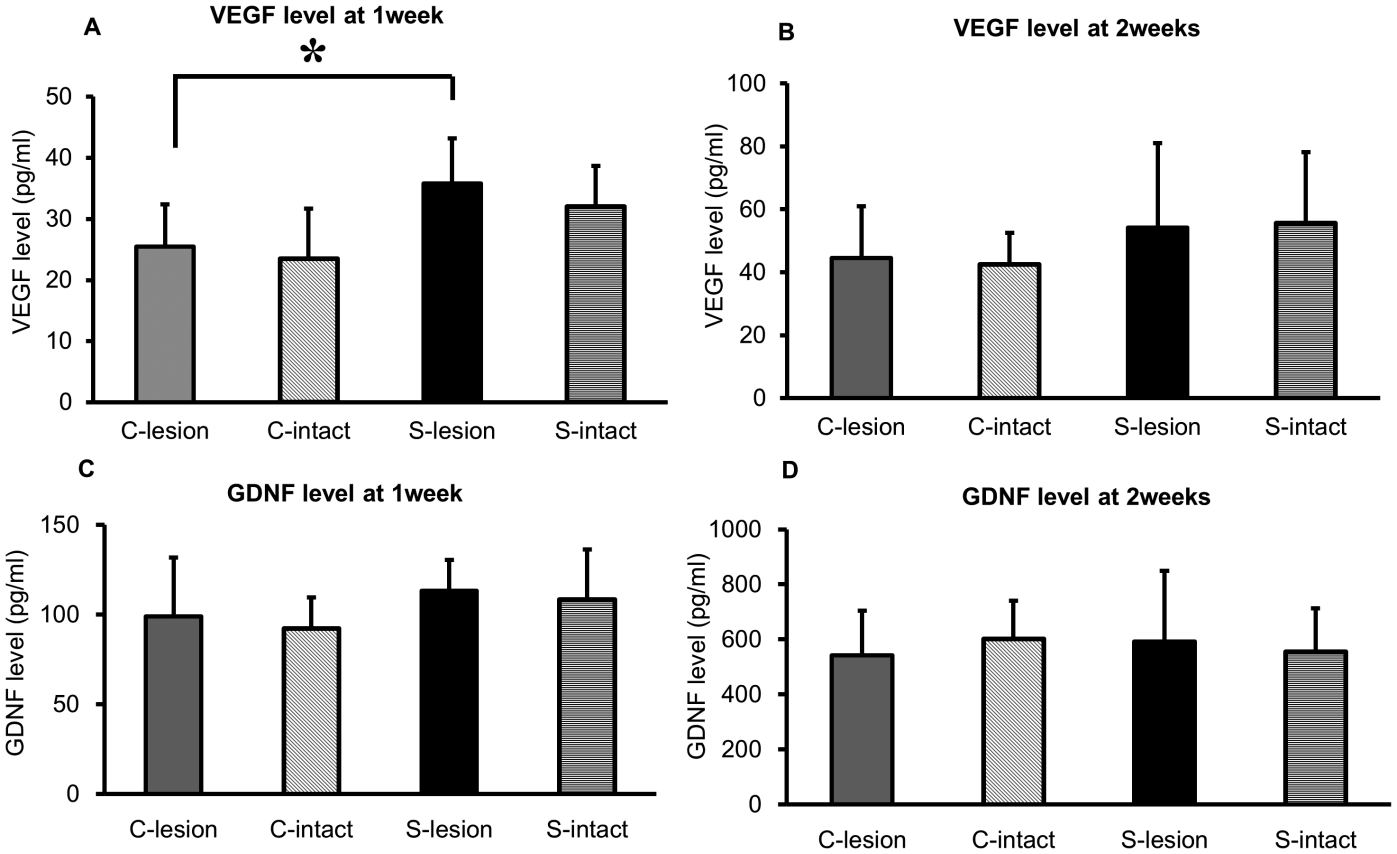
Results of ELISA analysis for VEGF and GDNF. (A, B) In the lesioned striatum, VEGF was significantly increased by SCS at 1 week after 6-OHDA lesion (*p<0.05). At 2 weeks after 6-OHDA lesion, VEGF level in the lesioned striatum also appeared elevated, but did not reach statistical significance. (C, D) GDNF in the striatum of both sides was not significantly increased by SCS at 1 and 2 weeks after 6-OHDA lesion. (C-lesion: control group lesioned side Striatum; C-intact: control group intact side Striatum; S-lesion: 50 Hz SCS group lesioned side Striatum; S-intact: 50 Hz SCS group intact side Striatum, n = 10, respectively).

VEGF level of the lesioned striatum in rats that received 50 Hz SCS increased compared to that of control group at 1 and 2 weeks after 6-OHDA lesion, respectively (50 Hz: 35.8±7.4 pg/ml and 54.3±26.8 pg/ml; control group: 25.5±6.9 pg/ml and 44.6±11.6 pg/ml at 1 and 2 weeks, respectively, Fig. 5A and B) and reached statistical significance at 1 week after 6-OHDA lesion (ANOVA; F (3, 42); p = 0.0006; p value<0.05, Fig. 5A). On the other hand, 50 Hz SCS did not increase GDNF level in the striatum at 1 and 2 weeks after 6-OHDA lesion compared to that of rats in control group (50 Hz: 113.3±17.1 pg/ml and 592.4±256.3 pg/ml; control group: 99.0±32.8 pg/ml and 542.5±161.3 pg/ml, at 1 and 2 weeks, respectively, Fig. 5C and D).

## Discussion

The present study showed that SCS exerted neuroprotective effects on PD model of rats characterized by behavioral and immunohistochemical amelioration. The neuroprotective effects of 50 Hz SCS appeared optimal, compared to those of 2 Hz and 200 Hz SCS. VEGF level increased in the lesioned striatum of rats that received 50 Hz SCS, implicating that the neuroprotective effects of 50 Hz SCS may partially involve a VEGF-mediated mechanism.

### SCS parameter

There are few reports about the effect of SCS for animal model of PD, and therefore there is almost no report that would be helpful for our choice of electrical parameter. For this reason, we had chosen the parameters referring to the report of electrical stimulation experiments for the various central nervous diseases. In the previous report about the effect for the cerebral blood flow, 50 Hz SCS had the highest increasing effect of the cerebral blood flow [23]. In reference to this report, the other majority of SCS experiments about the cerebral blood flow are used 50 Hz SCS. On the other hand, according to our electrical stimulation experiments on cerebral infarction model of rats, we had reported that low frequency stimulation (i.e. 2 or 10 Hz) were effective for reduction of volume of cerebral infarction [12]. Furthermore, 333 Hz SCS are used in the report of the effect of SCS for PD model [2]. Referring to these reports, we had selected these three stimulation frequencies (i.e. 2, 50, and 200 Hz). It has been reported that 200 or 2000 Hz SCS had no increasing effect in cerebral blood flow [24]. We have considered that this result is a one of the reason why 200 Hz SCS hasn’t obtained good effect in this study. We also have impression that the 200 Hz SCS might damage to rat spinal cord, and rat itself.

### Current status of SCS

In clinical practice, SCS was introduced in 1967 for the treatment of chronic intractable neuropathic pain [25]. Today, SCS targeting the dorsal column is clinically used as a valuable treatment for neuropathic pain, especially for failed back surgery syndrome (FBSS) [26], complex regional pain syndrome type 1 [27]. The mechanisms of pain relief have not yet been well elucidated. Previously, the gate control theory advanced the notion that pain signals from the peripheral nerve were presynaptically inhibited in the spinal cord [28]. However in recent years, other neurochemical factors and electrophysiological factors have been thought to mediate pain [29]–[36]. On the other hand, the increase of cerebral blood flow has been shown to accompany the therapeutic effects of SCS [37]–[39]. In animal experiments, several studies have demonstrated efficacy of SCS in models of cerebral infarction or vasospasm after subarachnoid hemorrhage [3]–[10]. SCS was also used for the treatment against cardiac ischemia [40], or postoperative ileus [41]. The mechanism of vasodilation by SCS appears to be related to suppression of sympathetic activity [42], [43], as well as indirect activation of the brainstem or cerebellar vasomotor centers [6], [7], [44], and/or causing the release of rapid vasoactive substances such as nitric oxide or calcitonin gene-related peptide [45]–[47]. Moreover, SCS was accompanied by dilation of small arteries in the subarachnoid space without visible changes of intraparenchymal vessels in diameter [4], altogether supporting the concept of a humoral effect by SCS. Accordingly, assessment of alterations in the vascular system (i.e., monitoring VEGF levels as in the present study) may provide insights into the neuroprotective effects of SCS.

### Neurotrophic and growth factors and electrical stimulation

Electrical stimulation has been used in the clinical setting for various diseases of the central nervous system, including epilepsy, central pain, and psychological disorders like schizophrenia and depression. Electrical stimulation of the cerebral cortex increased the expression of neurotrophic and growth factors, such as GDNF, BDNF and VEGF [12]. Previously we demonstrated that parenchymal stimulation exhibited significant upregulation of GDNF and VEGF for chronic-phase ischemic stroke model of animals [13]. And in PD rats, stimulation of subthalamic nucleus increases BDNF in nigrostriatal system [16]. However, there is almost no report on the relationship between SCS and neurotrophic and growth factors. Very recently, Yadav and colleagues demonstrated the possibility that high thoracic SCS might have neuroprotective effects for PD model of rat, and pronounced that SCS might increase production or delivery of neurotrophic factors [11]. In our study, we demonstrated that VEGF increased in the lesioned striatum of rats that received SCS. SCS didn’t upregulate BDNF (data not shown). As previously reported, SCS may increase cerebral blood flow and enhance patency of the cerebral microvasculature [3]–[10], again invoking the effects of SCS on the vascular system. VEGF is known to enhance glial proliferation and angiogenesis with synergistic neuroprotective effects [48]–[51]. Neuroprotective effects have also been associated with neurogenesis and intrinsic neurorestoration [50], [52]–[53]. Furthermore, increased VEGF signaling may result in neuroprotective effects thereby enhancing the survival of dopaminergic neurons, which suggests a potential therapeutic application for PD [49]–[50], [54]. Additionally, VEGF may protect dopaminergic neurons by improvement of microcirculation through enhanced angiogenesis. These multi-pronged vasculature-based neuroprotective pathways might have been elicited by the observed VEGF elevation following SCS treatment in our PD animals.

### SCS and Parkinson’s disease

In 2009, Fuentes and co-workers reported that SCS restores locomotion in animal models of PD [2], indicating that SCS may alleviate PD-related akinesia. It is known that neural fibers which are most activated by SCS might be the superficial fibers of the dorsal columns, although the underlying mechanisms are still not well understood. SCS may facilitate corticostriatal oscillatory mode of neuronal activity with subsequent increase of locomotion [2]. However, a clinical study showed that SCS failed to relieve akinesia or restore locomotion in PD [55]. This discrepancy may be due to the limitation of the PD model in approximating the clinical pathophysiology, as well as the differences in SCS stimulation parameters in the laboratory and the clinic. In the present study we showed that 50 Hz SCS may afford neuroprotective effects on the nigrostriatal system of PD rats. Yet, it has translational limitations as patients receive SCS after PD. Yadav and colleagues demonstrated that SCS had neurorestorative effect. In their protocol, SCS started one week after 6-OHDA administration [11]. We must conduct the additional studies, which demonstrate the neurorestorative effects of SCS on PD model of rats in the future.

## Conclusions

This study demonstrates that high cervical SCS exerts neuroprotective effects in PD model of rats by increasing VEGF levels in the lesioned striatum. SCS is supposed to suppress or delay PD progression. In the future, SCS may become a less invasive therapeutic option for PD patients, although further preclinical experiments are warranted to confirm the efficacy, safety, and mechanisms of action.
